# Time for a full digital approach in nephropathology: a systematic review of current artificial intelligence applications and future directions

**DOI:** 10.1007/s40620-023-01775-w

**Published:** 2023-09-28

**Authors:** Giorgio Cazzaniga, Mattia Rossi, Albino Eccher, Ilaria Girolami, Vincenzo L’Imperio, Hien Van Nguyen, Jan Ulrich Becker, María Gloria Bueno García, Marta Sbaraglia, Angelo Paolo Dei Tos, Giovanni Gambaro, Fabio Pagni

**Affiliations:** 1 Department of Medicine and Surgery, Pathology, Fondazione IRCCS San Gerardo dei Tintori, Università di Milano-Bicocca, Monza, Italy; 2 Division of Nephrology, Department of Medicine, University of Verona, Piazzale Aristide Stefani, 1, 37126 Verona, Italy; 3 Department of Pathology and Diagnostics, University and Hospital Trust of Verona, P.le Stefani n. 1, 37126 Verona, Italy; 4 Department of Medical and Surgical Sciences for Children and Adults, University of Modena and Reggio Emilia, University Hospital of Modena, Modena, Italy; 5 Department of Electrical and Computer Engineering, University of Houston, Houston, TX 77004 USA; 6 Institute of Pathology, University Hospital of Cologne, Cologne, Germany; 7 VISILAB Research Group, E.T.S. Ingenieros Industriales, University of Castilla-La Mancha, Ciudad Real, Spain; 8 Department of Pathology, Azienda Ospedale-Università Padova, Padua, Italy; 9 Department of Medicine, University of Padua School of Medicine, Padua, Italy

**Keywords:** Machine learning, Artificial intelligence, Image analysis, Nephropathology, Classification, Segmentation

## Abstract

**Introduction:**

Artificial intelligence (AI) integration in nephropathology has been growing rapidly in recent years, facing several challenges including the wide range of histological techniques used, the low occurrence of certain diseases, and the need for data sharing. This narrative review retraces the history of AI in nephropathology and provides insights into potential future developments.

**Methods:**

Electronic searches in PubMed-MEDLINE and Embase were made to extract pertinent articles from the literature. Works about automated image analysis or the application of an AI algorithm on non-neoplastic kidney histological samples were included and analyzed to extract information such as publication year, AI task, and learning type. Prepublication servers and reviews were not included.

**Results:**

Seventy-six (76) original research articles were selected. Most of the studies were conducted in the United States in the last 7 years. To date, research has been mainly conducted on relatively easy tasks, like single-stain glomerular segmentation. However, there is a trend towards developing more complex tasks such as glomerular multi-stain classification.

**Conclusion:**

Deep learning has been used to identify patterns in complex histopathology data and looks promising for the comprehensive assessment of renal biopsy, through the use of multiple stains and virtual staining techniques. Hybrid and collaborative learning approaches have also been explored to utilize large amounts of unlabeled data. A diverse team of experts, including nephropathologists, computer scientists, and clinicians, is crucial for the development of AI systems for nephropathology. Collaborative efforts among multidisciplinary experts result in clinically relevant and effective AI tools.

**Graphical abstract:**

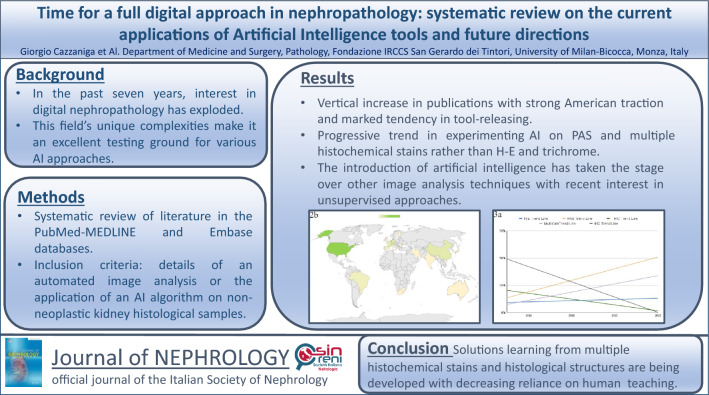

## Introduction

In recent years, the growing digitalization of pathology departments has led to an exponential diffusion of scanners and increased use of whole slide images (WSIs) instead of or complementing traditional glass slides, with a broad improvement of teleconsultation, education, and image archiving [1]. Simultaneous progress in Machine Learning (ML) and hardware allowed the integration of artificial intelligence (AI) in pathology, leading to the development of computer-aided tools for tasks previously performed manually, and assisting pathologists in the time-consuming work of image analysis [2, 3]. One of the most intriguing digital areas is nephropathology [4], a sub-specialty dealing with low-incidence disease. Nephropathology has a long history of integrative approaches which goes back to 1979, when grayscale tissue thresholding was performed to capture a histological section of the kidney [5]. Since then, image analysis in nephropathology has been driven by multidisciplinary teams including nephrologists, biologists, pathologists, and computer scientists, leading to ongoing cross-disciplinary exchange and enrichment of the knowledge and skills of each professional involved [6, 7]. Renal pathology is a peculiar niche where a variety of histological techniques are performed routinely to reach the final diagnosis. In light microscopy, a variety of stainings traditionally including hematoxylin and eosin (H-E), periodic acid-Schiff (PAS), trichrome (TRIC) and silver staining are used to yield a range of recently clarified descriptors [8] in the respective tissue compartments, a task that can be handed to ML systems [9]. However, the need for different tools for the digitization of congo-red or crystal birefringence, immunofluorescence and electron microscopy, makes full computational integration very challenging [10, 11]. Furthermore, in the field of transplantation, histological evaluation of the donor kidney frequently includes the use of immunohistochemistry (IHC), which is integrated into the scoring system to assess features of rejection, adding an additional layer of complexity to the process [12]. So, several different tasks can be asked of a ML algorithm on kidney biopsy slides, ranging from simple tasks like counting glomeruli, quantifying IHC or segmenting areas of interstitial fibrosis to complex ones like classification of glomerular lesions, where the overlap of clinical and histological patterns requires higher computational power, greater amount of data and an efficient and smarter workflow. In this review, a comprehensive outline is provided on the historical development of image analysis and the utilization of artificial intelligence in nephropathology. Emphasis is placed on the current state of the art, and how it is addressing the challenges posed by increasing task complexity and the demand for handling large amounts of data.

## Materials and methods

Our study followed the Meta-analysis of Observational Studies in Epidemiology (MOOSE) guidelines for conducting a systematic retrieval of literature [13]. The study was registered on the Open Science Framework (OSF) database with the following https://doi.org/10.17605/OSF.IO/YXUW4.

### Search strategy and inclusion/exclusion criteria

We systematically searched for relevant studies in the PubMed-MEDLINE and Embase databases using a comprehensive search strategy and applying specific inclusion and exclusion criteria (Table 1). Briefly, inclusion criteria were availability of the details of an automated image analysis or the application of an AI algorithm on non-neoplastic kidney histological samples. Publications with abstracts alone were excluded, as were reviews and published letters to the editor with no original data and image analysis methods. The search was conducted up to August 15th, 2022.

**Table 1:** Search strategies

*Pubmed*
#1 "image"[Title/Abstract] AND "analysis"[Title/Abstract] #2 "artificial"[Title/Abstract] AND "intelligence"[Title/Abstract] #3 "morphometry"[Title/Abstract] OR "morphometric"[Title/Abstract] OR "histomorphometric"[Title/Abstract] OR "AI"[Title/Abstract] OR "algorithm*"[Title/Abstract] OR "neural network"[Title/Abstract] OR "neural networks"[Title/Abstract] OR "convolutional"[Title/Abstract] OR "deep-learning"[Title/Abstract] OR "deep-learning"[Title/Abstract] OR "computational"[Title/Abstract] OR "computerized"[Title/Abstract] OR "automated"[Title/Abstract] OR "machine-learning"[Title/Abstract] OR "machine-learning"[Title/Abstract] #4 #1 OR #2 OR #3 #5 "kidney"[Title/Abstract] OR "renal"[Title/Abstract] #6 "transplant*"[Title/Abstract] OR "graft*"[Title/Abstract] OR "allograft*"[Title/Abstract] #7 #4 AND #5 AND #6 #8 "Artificial Intelligence"[Mesh]) AND ("Kidney Transplantation"[Mesh] #9 #7 OR #8
*Embase*
#1 image AND analysis #2 artificial AND intelligence #3 morphometry OR morphometric OR histomorphometric OR AI OR algorithm* OR “neural network” OR “neural networks” OR convolutional OR “deep learning” OR deep-learning OR computational OR computerized OR automated OR “machine learning” OR machine-learning #4 #1 OR #2 OR #3 #5 kidney OR renal #6transplant* OR graft* OR allograft* #7 #4 AND #5 AND #6

No study type filters nor language restrictions were applied. References listed in all identified studies were also hand-searched to retrieve potential additional studies. Initial screening of articles by title/abstract was performed with the aid of the online systematic review web-app QRCI [14]. Two independent reviewers (GC and MR) determined the eligibility of published studies, with any disagreements resolved through consensus. The two authors independently extracted data from the included studies using a standardized form.

### Data extraction

Extracted data included: authors’ names, publication year, country of study, area of specialization of the first author (pathologist, nephrologist, computer scientist or other), if the study was conducted on native or transplanted kidneys, technique used in the study (light microscopy, immunofluorescence, electron microscopy or other), staining of the slides (H-E, PAS, TRIC or multi-stain), histological region of interest (glomerulus, tubulo-interstitium, vascular compartment or whole slide), image analysis tasks or AI algorithms (quantification, detection, segmentation or classification), type of learning (supervised, unsupervised or other), if a paper-related free-to-use tool was released after publication. The term “quantification” was reserved for those cases in which an automated image analysis was performed without integration of AI (i.e. pixel-counting or rules-based approach). Statistics were extracted with Excel 2016 (Microsoft, Redmond, WA, USA) in order to analyze the trend of the various parameters and create plots and charts. Column charts were created listing publications after 2015 for easier and more informative visualization given the low number of studies done in previous years.

## Results

**Fig. 1:**
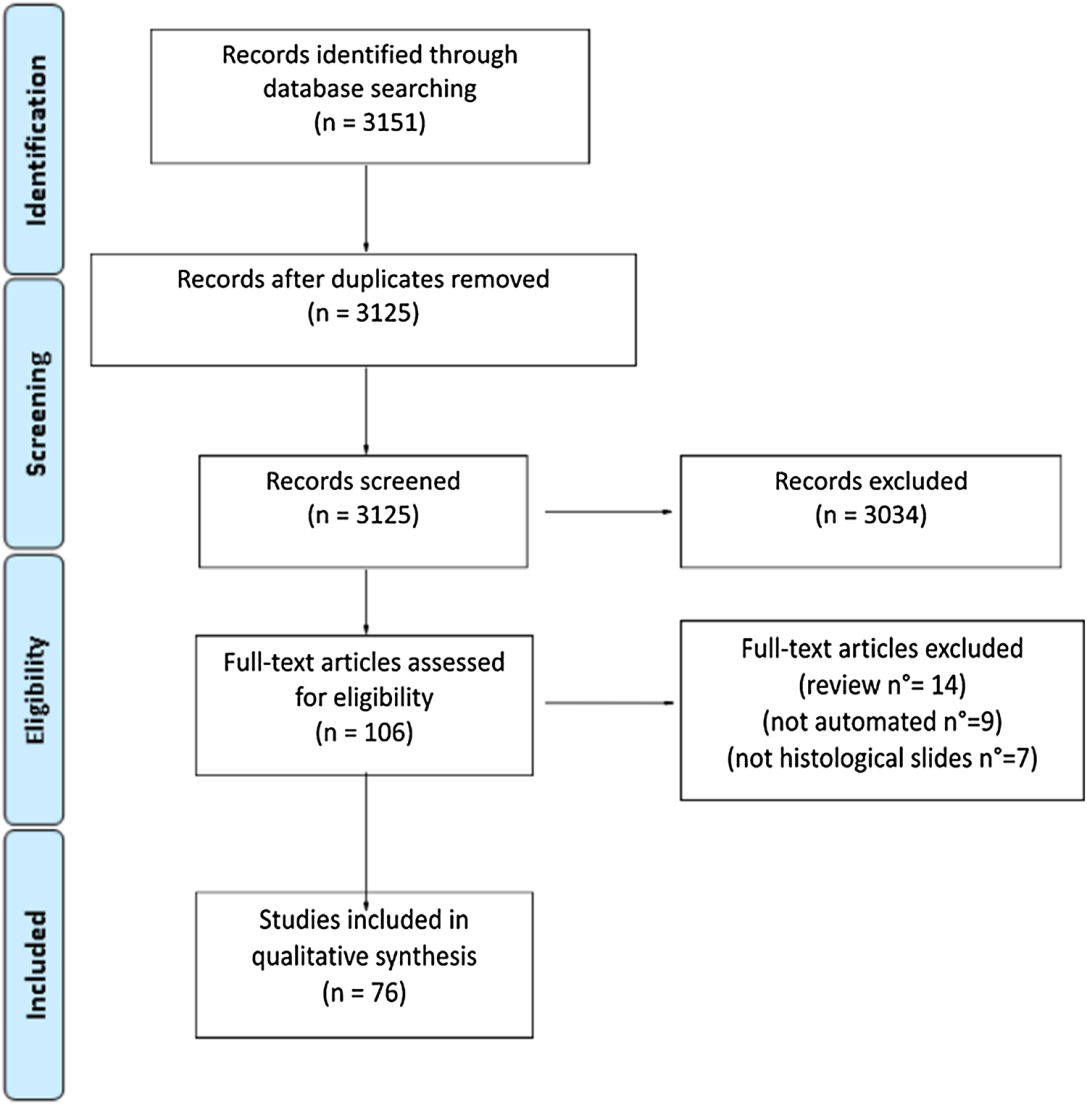
Review flowchart

**Fig. 2:**
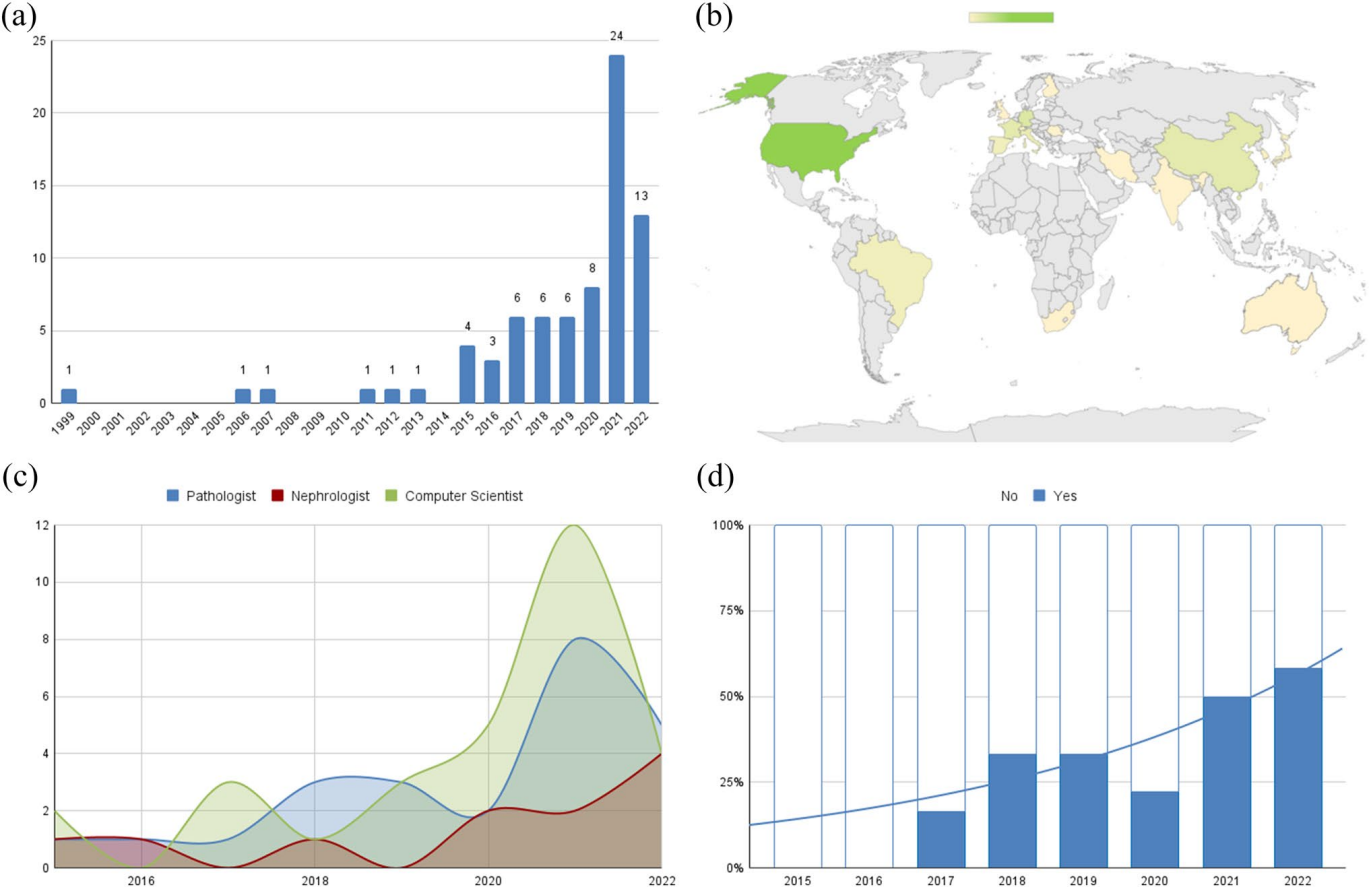
**a** Publications per year: bar chart showing the strong increase in publications in recent years; **b** World heat-map showing the strong American leadership in this field of research; **c**: professional profiles testing AI in nephropathology. Computer scientists have emerged in recent years as fundamental contributors in the field of digital nephropathology; tool release per year; the increasing tendency to release useful and free-to-use tools was included in more than 50% of publications in 2022 (until August)

Of the 3151 abstracts screened, 26 duplicates and 3034 articles lacking relevance to the topic were excluded; the full text was available for each remaining article. Based on the full-text screening, additional restrictions were applied (Fig. 1) until obtaining a final set of 76 original research articles. The majority were produced in the previous 7 years, and nearly half of them in the last 2 years Fig. 2a. Thirty-two articles were produced in the United States, 6 in Germany, 5 in France and China, 4 in Italy, 3 each in Brazil, Netherlands and Spain, 2 each in Switzerland, Japan and South Korea and 1 each in the UK, Taiwan, Australia, India, Finland, Romania, Iran, South Africa and Austria. The geographic distribution of the literary production is shown in Fig. 2b.

### Professional profiles and integration of tools

Computer scientists were the main contributors to the output in this area (31 papers, 40.8%), followed by pathologists (26 papers, 34.2%) and nephrologists (13 papers, 17.1%), while other professionals carried out the research only occasionally (6 papers, 7.9%). Figure 2c shows the chronological trend of the professional figures most involved in scientific production in the field of digital nephropathology. Some of the works (27 papers, 36%) were followed by the release of free-to-use tools, available either in the form of downloadable software, codes or pre-trained models. This practice is spreading more and more; over the last few years more than half of the works published led to the release of a tool (Fig. 2d).

### Technical setting

**Fig. 3:**
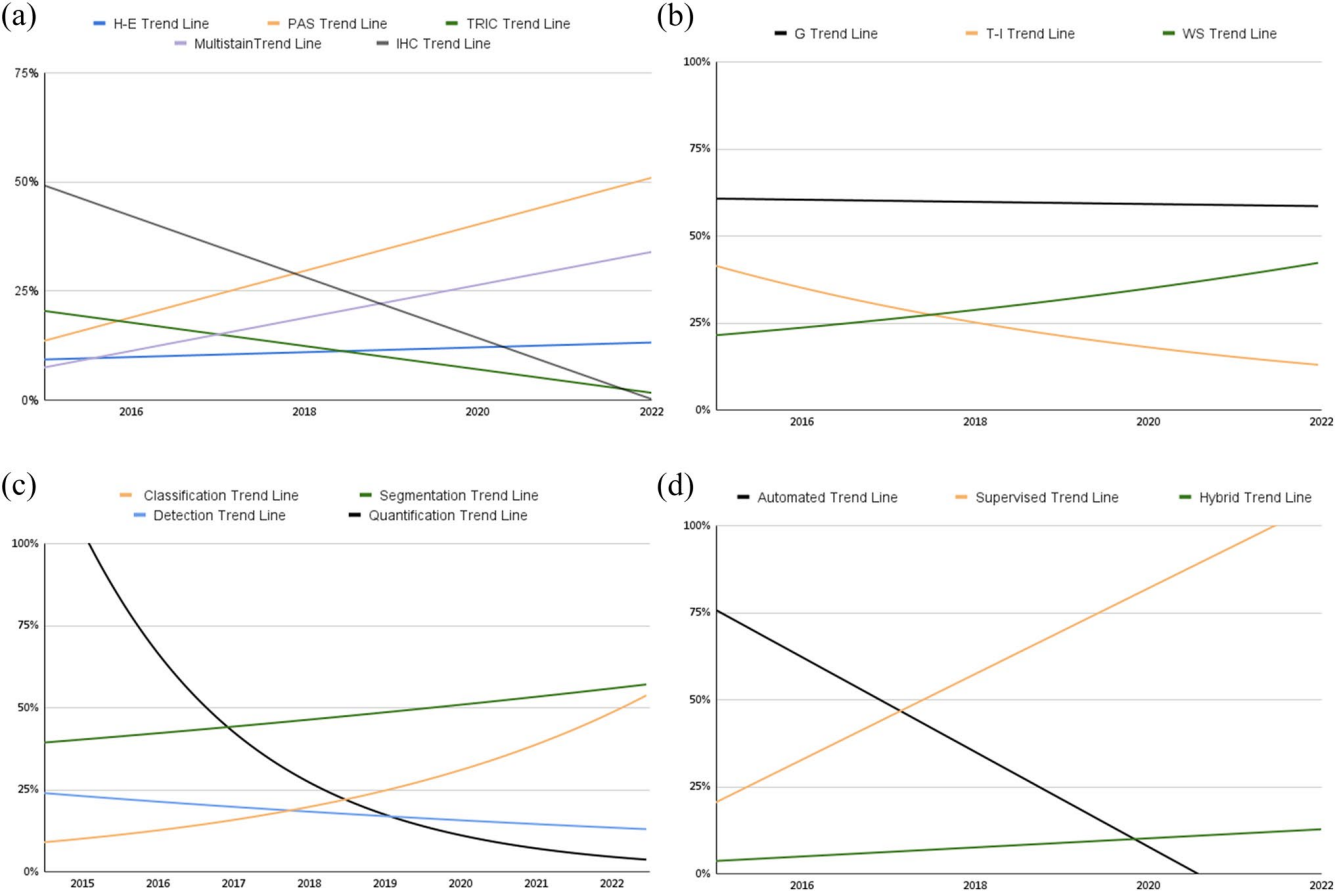
**a** Staining per year. Line chart showing linear functions of staining used over time. Notably, it can be seen that the most recently used stain is PAS and that there is growing interest in multi-stain approaches; **b** kidney structures per year. Exponential line chart of kidney structure focus over time showing the increasing interest in multi-structure approaches; **c** tasks per year. Exponential increase in interest for classification tasks and decrease in detection and quantification algorithms; **d** learning approaches per year. Line chart showing linear function of image-analysis approach over time. Interest in automated image-analysis without the help of AI has rapidly decreased in favor of AI-based approaches. In recent years, hybrid and unsupervised approaches have slowly started spreading in this field

Only 7 works (9%) used techniques other than light microscopy (69, 91%). In particular, four works are on immunofluorescence, one on electron microscopy, one on lightsheet microscopy and one on the 3D reconstruction of glomerulus. The stains used in light microscopy were subdivided fairly evenly, with many experiments being performed on H-E (9 papers, 13%), PAS (23 papers, 33%), trichrome (7 papers, 10%), IHC (18 papers, 26%), combined approaches (10 papers, 15%) and 1 (1.5%) experiment each for Congo-Red and Toluidine Blue. There has been increased interest for PAS in recent years, as shown in (Fig. 3a).

### Field of application: native vs transplant

Research has mostly focused on native kidneys (62 papers, 82%), with considerable analyses being performed on transplanted kidneys (14 papers, 18%); some works focused on a single pathology (8, 6%). More precisely, four works are on IgA nephropathy, two on lupus nephritis and two on diabetic nephropathy, while the majority cross-sectionally explored renal pathology and glomerular diseases.

### Structures of the kidney and AI tasks

Most of the studies focused on the glomerulus (41 papers, 54%), fewer on tubulointerstitium (9 papers, 12%), vascular compartment (1 paper, 1%) and immune cells (1 paper, 1%), while 20 works (26%) focused on more than 1 structure (glomerulus and tubulointerstitium) or on the entire WSI. The latter recorded a notable increase in interest at the expense of the tubulointerstitial compartment, while interest in the glomerulus remained stable (Fig. 3b).

Of the 76 manuscripts analyzed, one focused on stain transformation in nephropathology, one on 3D reconstruction of the glomerulus and one on quality control of kidney sample slides. Some of the typical experiments carried out in nephropathology included quantification without the use of AI (14 papers, 18%), mainly in the early years of image analysis, and others with the use of AI, such as detection (8 papers, 11%), segmentation (31 papers, 41%), and classification (20 papers, 26%). The trend recorded in recent years is shown in (Fig. 3c).

### Learning approaches

With regard to image analysis in the nephropathological community, three approaches have been tested: an automated approach without the use of AI, a supervised ML approach, and a “hybrid” supervised/unsupervised or fully unsupervised ML approach. The automated approach, which involves manually adjusting settings to carry out an automated process, was tested in only 18% of papers (14 out of 52); the supervised ML approach was the most popular, involving 68% of papers (52 out of 76) focusing on this; the hybrid or unsupervised ML approach also gained some attention, with 9% of papers (7 out of 76 total) (Table 2), with a slight but significant increase in interest in recent years (Fig. 3d).

**Table 2:** Evolution of Unsupervised and Hybrid Supervised-Unsupervised approach in nephropathology

Author	Year	Country	Journal	Learning approach	Stain	Task	Architecture
Yao [15]	2022	USA	*J Med Imaging Bellingham*	Self Supervised	PAS	C	ResNet-50
Bouteldja [16]	2022	Germany	*J Pathol Inform*	Self Supervised	PAS, IHC	ST	U-GAT-IT
Mascolini [17]	2022	Italy	*BMC Bioinform*	Self Supervised	IF	C	NASNet
Murphy [18]	2022	USA	*Bioinformatics*	Self Supervised	IHC	C	DenseNet-121
Lee [19]	2022	USA	*Sci Rep*	Unsupervised	Trichrome	S	DeepLabV3 + & ResNet-18
Sato [20]	2021	Japan	*Kidney Int Rep*	Unsupervised	HE	C	NASNet
Gadermayr [21]	2019	Austria	*IEEE Trans Med Imaging*	Unsupervised	PAS	S	CycleGAN

*PAS* Periodic-acid-Schiff, *IF* immunofluorescence, *HE* hematoxylin–eosin, *C* classification, *ST* stain transformation, *S* segmentation

## Discussion

Digital pathology solutions may be of particular interest in the field of renal disease diagnosis for routine purposes such as the creation of expert networks and for the research field with the application of AI. Its unique multidimensional nature makes it an ideal area for integration with image analysis from multiple perspectives. However, this variability, along with a significant increase in research publications over the past 2 years, makes it challenging to monitor trends accurately. Therefore, it is crucial to trace a timeline of research development to better comprehend the current state of the art and identify future directions [22].

Informatics tools in pathology have evolved to varying degrees around the world since the infancy of informatics in pathology in the 1950s [23]. The exponential growth of this field driven by AI in the last 5 years has, however, highlighted the need for a “new” type of pathologist with adequate training in computer science, as well as the support of pathology computer scientists who can translate new ideas into clinical practice [24]. Successfully implemented training programs in pathology residency already exist, but they are a prerogative of very few universities mostly located in the USA [7]. To fully leverage the capabilities of AI, nephropathologists need access to various AI tools for different diagnostic tasks. Both commercial and open-source next-generation AI tools have become available in recent years. It is essential to acknowledge that, until now, no AI tools have obtained official approval from healthcare regulatory agencies for diagnostic purposes in nephropathology. Consequently, the utilization of AI tools in this field is predominantly experimental and research-driven. Despite this, some applications could soon successfully be used in routine practice, such as the use of a deep learning-based transformation of H&E stained tissues into other special stains, with important effects on laboratory standardization and turnaround time (TAT). Moreover, the development of software solutions that can integrate multiple AI tools into a single platform or interface is a promising step towards making these tools more widely available and accessible to healthcare professionals in the future. Proposed solutions include creating software that integrates several functions into one kit (for instance, with the creation of a kit that integrates the ability to detect, segment, and classify glomeruli) [15] and developing apps like "EMPAIA App Interface'' that can integrate AI tools into pathology workstations through widely-used web communication protocols and containerization [25].

Renal biopsies require special stains beyond H-E, leading to heterogeneity in datasets [26]. Deep learning, using deep convolutional neural networks is capable of identifying patterns in complex and heterogeneous histopathology data [27]. Image analysis quantification was initially performed on images with higher color contrast such as hematoxylin-DAB, for the detection of cells, or trichrome stain, for the detection of interstitial fibrosis. Most studies obtained good results on homogeneous datasets with a single staining, particularly in image segmentation [28–30]. The increase in computational capabilities and the interest in a comprehensive assessment of renal biopsy have led to an explosion of research on approaches that rely on two or more stains. Four-stain analysis showed that PAS stain achieved the best results, mainly due to less inter-laboratory variability and superior definition of boundary by highlighting basement membranes, which in turn provides superior definition of the boundary and easier segmentation tasks [31]. However, some convolutional neural networks performed better when different stains were used simultaneously for training instead of using a single stain: the authors attributed these results to the fact that many kidney pathologies are focal, and only observed in a specific section [32]. Innovative approaches to slide staining in nephropathology include virtual staining using hyperspectral imaging [33], quantitative phase imaging [34], and autofluorescence [35, 36], enabling the possibility of multiple stains upon a single slide and the computational transformation of an already stained WSI into another stain [37].

Since the Banff conference in Pittsburgh in 2019, where the potential use of AI and ML algorithms in solid organ transplantation was discussed, digitization and standardization have become increasingly pressing concerns in this field [38, 39]. To address this, a working group called the Digital Pathology Banff was formed to define standards to ensure a smooth and effective transition [32, 40, 41], while some tools providing reproducible, quantitative specific parameters, like tubulo-interstitial injury (ci, ct, ti, i,t, i-IFTA and t-IFTA parameters) [42, 43] or automatic detection for C4d (C4d parameter) [44] were developed. Other research has focused on using classification algorithms to categorize transplant biopsies into one of three categories: normal, rejection, or other diseases [32], while a smaller number of studies aimed at evaluating the pre-transplant kidney rather than evaluating rejection [12]. In this context, converting frozen tissue sections to be similar to formalin-fixed and paraffin-embedded ones can rectify cryosection artifacts while preserving clinically relevant features, resulting in significant improvements, as is done in other settings [45].

In most of the publications before 2017, the year when ML made its way into nephropathology, automated image analysis consisted mainly in thresholding and color image segmentation algorithms, aimed at detecting and quantifying pixels and areas with specific staining features [46]. Indeed, these experiments focused on slides in which the stains proposed a large image contrast such as trichrome (mostly for assessment of tubulo-interstitial fibrosis) and IHC [47–49]. No article reported the use of ML on kidney histological samples before that year, but it must be emphasized that in 1999 a simple single-layer perceptron network was designed and trained with features extracted from about 100 kidney transplant biopsies to predict the final diagnosis [50]. Initially, the main efforts were made to perform structure recognition through semantic segmentation, that is labeling each pixel in an image to outline object boundaries [51]. Although segmentation alone may be of limited use in routine practice, it lays the groundwork for performing more sophisticated tasks [46]. Finally, a multiclass segmentation model made its first appearance in 2019 as a single-stain model and was later followed by a multi-stain one [31, 52]. The glomerulus has been the primary target also for classification, which refers to a predictive modeling problem where a class label is assigned to an object: classifying glomerular lesions is a fundamental step toward the diagnosis of many kidney diseases, and AI has the potential to be a support tool for pathologists, thereby decreasing interobserver variability [53]. The first efforts focused on the application of well-known approaches such as k-nearest-neighborhood or convolutional neural networks, but since 2020 most works have integrated an automated computational pipeline by WSI. Gradually, improvements in detection, segmentation and feature extraction better prepared the field for the final classification task, which involves integrating two or more ML models into a fully-automated workflow (i.e., one for features extraction and one for classification) [30, 32, 54].

**Fig. 4:**
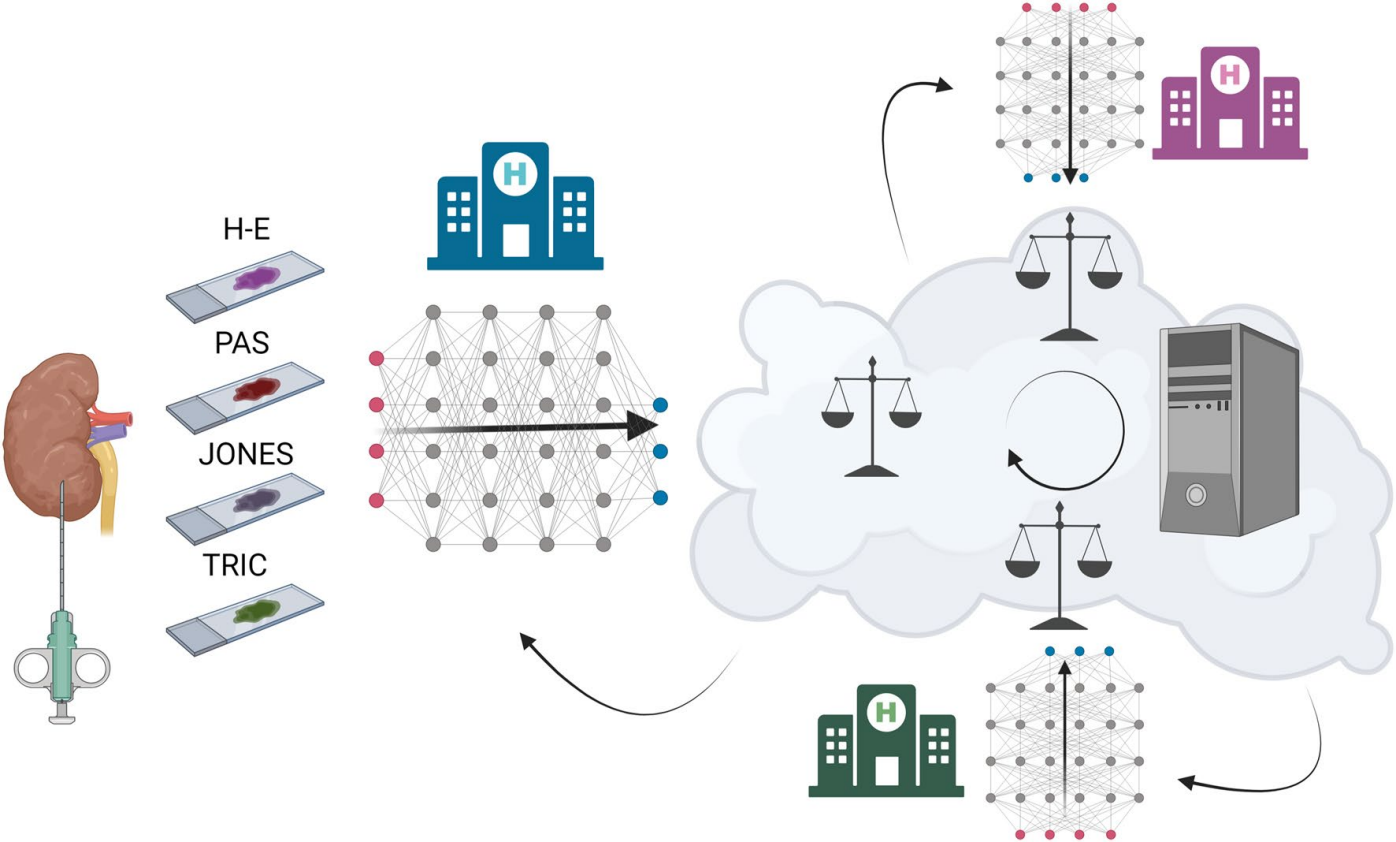
Federated learning in nephropathology. Each institution (node) trains the model using local data, and sends back model weights to the central server or to other institutions (peer-to-peer) without the transfer of slides. This will allow for pooling of greater amounts of data, therefore pushing towards a faster transition to increasingly complex multi-stain and multitask algorithms with hybrid or unsupervised approaches

The main type of learning tested on kidney tissue is the supervised kind, where models are fit on training data consisting of inputs and usually expert-labeled outputs. This approach relies on large sets of labeled image data, and is feasible when data sets come from institutions where pathologists manually annotate a certain number of slides for algorithm training. However, it becomes time-consuming, subject to occasional disagreement between pathologists, and difficult to apply when large amounts of data are required (e.g. for more complex tasks like the classification of overlapping features in glomerular lesions). For this reason, hybrid AI approaches in nephropathology, which adopt a large number of unlabeled images, have been tested [55, 56]. Despite being a largely unexplored field in pathology, our analysis shows an increase in interest in the application in the last 2 years. Recent approaches include self-supervised learning, where a model is trained on an auxiliary task for which ground-truth is available for free and web image mining, where large-scale unannotated images are achieved through online resources [15]. To overcome the difficulties of accessing and transmitting large amounts of data, a federated-learning model has been proposed for decentralized training [57, 58] (Fig. 4).

The rapidly increasing trend in digital nephropathology is poised to create new challenges and opportunities that will shape its future. With regard to computer vision and its application as machine learning in pathology, the development of standardized datasets will be critical for advancing research in nephropathology [59]. These datasets should be diverse and include a range of rare diseases to increase sample size and improve the accuracy of classifiers. While supervised training methods may be costly and time-consuming, semi-supervised or unsupervised methods may offer a more efficient approach by allowing architectures to find tissue representations better suited for the task at hand [60]. Incorporating clinical data into these datasets will also enhance the accuracy of classifiers. Furthermore, combining AI with other technologies such as biomarker discovery and "omics" will likely uncover new insights and improve diagnosis and treatment for patients with kidney disease, especially in an “in situ” approach [61]. A multi-dimensional view of tissue samples and precise extraction of molecular features are enhanced by WSI, especially in techniques such as matrix-assisted laser desorption/ionization, multiplex immunohistochemistry and digital spatial profiling, the former showing promising results in the non-neoplastic kidney like amyloid characterization or biomarker discovery in membranous nephropathy [62, 63].

## Conclusions

**Fig. 5:**
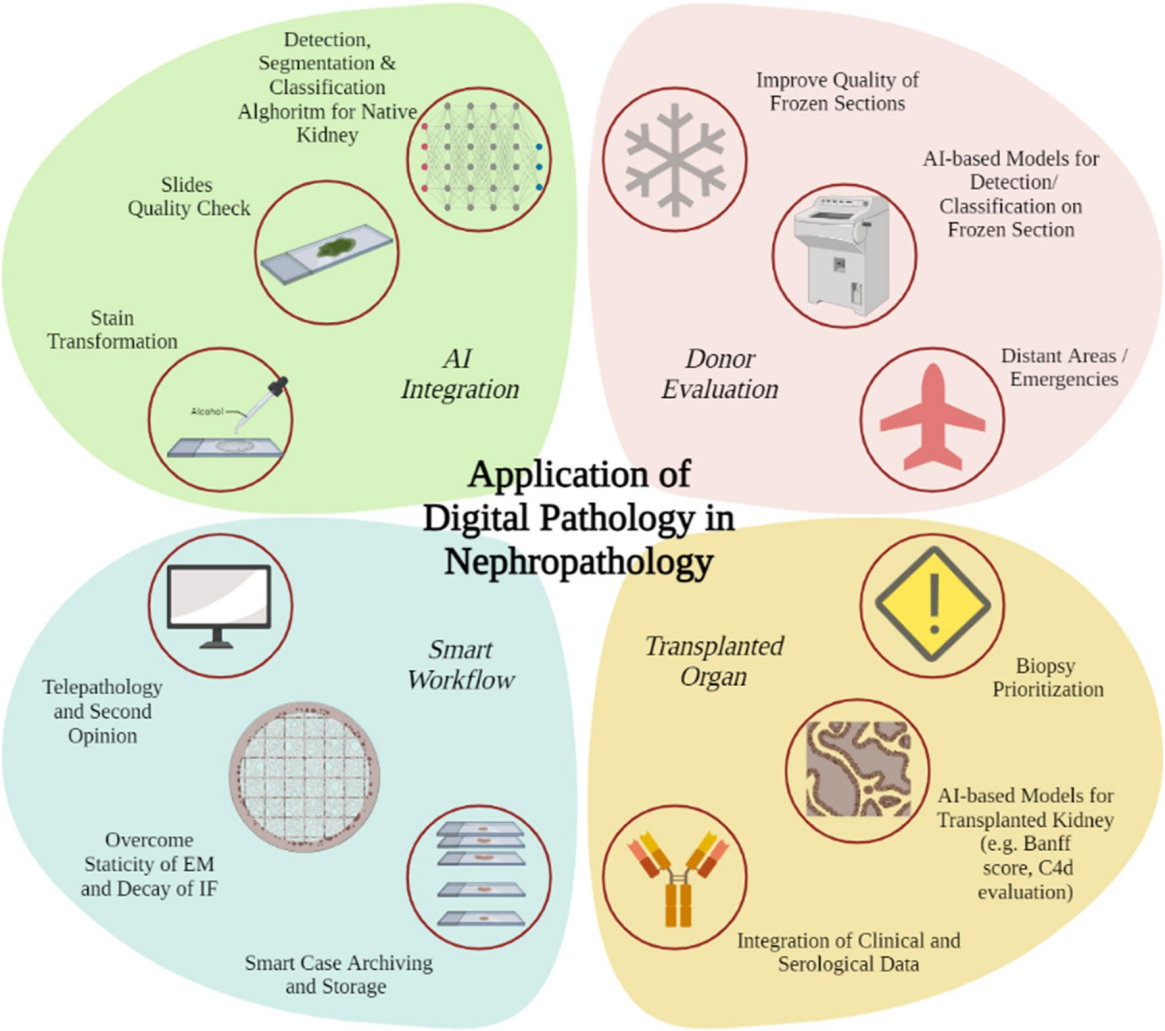
Application of digital pathology in nephropathology. In donor histological evaluation, where time plays a key role, digital pathology is used to connect centers and professionals in real time, and machine learning algorithms have been proposed to enhance images obtained from frozen sections [64] or interpret biopsy adequacy characteristics. In the field of transplant biopsy evaluation, AI “triage” classification of slides has been tested [32], as have automatic quantification of histological parameters of rejection and integration of histology into clinical and comprehensive algorithms [65]. Case storage and telepathology are two of the most widespread applications of digital pathology in nephropathology, a sub-specialty where the latter allows to overcome static Electron Microscopy (EM)  images and IF decay. The integration of AI in nephropathology opens the field to numerous applications ranging from, stain transformation, slide quality control and application of AI algorithms of image analysis

In the past 7 years, interest in nephropathology has exploded. This trend is expected to continue, and may revolutionize how nephrologists and pathologists approach kidney biopsy (Fig. 5). The unique complexities of nephropathology make it an excellent testing ground for various AI approaches that can create generalizable models for other research fields. With increasing computational capabilities and resources, solutions that integrate learning from multiple histochemical stains and histological structures, and consolidate different tasks, are being developed with decreasing reliance on human teaching.
